# Supplementary material for: Tracking individual honeybees among wildflower clusters with computer vision-facilitated pollinator monitoring
Source: PLoS One. 2021 Feb 11;16(2):e0239504. doi: 10.1371/journal.pone.0239504 (PMC7877608; doi:10.1371/journal.pone.0239504)
Supplement: S1 Table — (DOCX) [file pone.0239504.s001.docx]

# **S2 Table: Parameter settings used in experiments**

| **Parameter** | **Description** |
| --- | --- |
| Minimum Confidence | Minimum confidence with which YOLO identifies a given object |
| Minimum Frames | Number of frames after which background subtraction based detection is used in the video |
| Initial value of ${MDT}_{BS}$ ($d_{int}$) | Initial value for Maximum distance threshold (in pixels) for Background subtraction based detections (${MDT}_{BS})$ |
| Threshold value for exit probability $(\bar{\beta}$) | Threshold probability value at which the algorithm decides that the insect has left the frame |
| Minimum X | Leftmost boundary of the tracking area |
| Minimum Y | Topmost boundary of the tracking area |
| Maximum X | Rightmost boundary of the tracking area |
| Maximum Y | Bottommost boundary of the tracking area |
| Data threshold | Number of data points after which MDT are updated |
| Threshold value of background changes | Maximum number of changed regions within the calculated ${MDT}_{BS}$for background subtraction to be used as the detection method (otherwise deep learning is used) |
| Threshold number of undetected frames $(\bar{\tau})$ | Algorithm will predict the position of the insect after it has been undetected continuously for $\bar{\tau}$ frames |
| Minimum area covered by insect | Minimum area covered by the body of the insect (pixels) |
| Maximum area covered by insect | Maximum area covered by the body of the insect (pixels) |
| Minimum area for background change | Minimum area of a blob that must change for the software to treat the region as a change in background (pixels) |
| Maximum area for background change | Maximum area of a blob that changes for the software to treat the region as a change in background (pixels) using background subtraction before the algorithm switches to deep learning |

**Parameters used in Experiments**

| **Parameter** | **Parameters used for experiments** | | **Parameters used for example data analysis** | |
| --- | --- | --- | --- | --- |
|  | **Proposed Algorithm (HyDaT)** | **Deep Learning (YOLOv2)** | **Scaevola ground cover** | **Lamb’s Ear ground cover** |
| Minimum Confidence | 15 % | 15 % | 15 % | 15 % |
| Minimum Frames | 5 | - | 5 | 5 |
| $d_{int}$ | 40 | - | 40 | 40 |
| Threshold value for exit probability $(\bar{\beta}$) | 85% | 100% | 85% | 85% |
| Minimum X | Adjusted based on the insect to be tracked in each video file | Adjusted based on the insect to be tracked in each video file | 0 | 0 |
| Minimum Y |  |  | 0 | 0 |
| Maximum X |  |  | 1920 | 1920 |
| Maximum Y |  |  | 1080 | 1080 |
| Data threshold | 3 | 3 | 3 | 3 |
| Threshold value of background changes | 3 | - | 3 | 3 |
| Threshold number of undetected frames $(\bar{\tau})$ | 15 | - | 15 | 15 |
| Minimum area covered by insect (pixels) | 500 | - | 500 | 75 |
| Maximum area covered by insect (pixels) | 3000 | - | 3000 | 2000 |
| Minimum area for background change (pixels) | 50 | - | 50 | 50 |
| Maximum area for background change (pixels) | 8000 | - | 8000 | 8000 |
