# Supplementary material for: Tracking individual honeybees among wildflower clusters with computer vision-facilitated pollinator monitoring
Source: PLoS One. 2021 Feb 11;16(2):e0239504. doi: 10.1371/journal.pone.0239504 (PMC7877608; doi:10.1371/journal.pone.0239504)
Supplement: S1 Text — (DOCX) [file pone.0239504.s002.docx]

# **S3 Text: Data Collection details for experimental data analysis.**

Videos were recorded using a Samsung Galaxy S8 phone camera (12 MP CMOS sensor, f/1.7, $1920 \times1080$ pixels resolution,$60 fps$). Details on experimental setups are given in Table 1. Images were annotated with bounding boxes for YOLO using LabelImage [1] software.

**Table 1:** Experimental setup details and environmental conditions during the time of data for videos recorded in Scaevola and Lamb's-ear ground covers.

| **Parameter** | **Scaevola** | **Lamb's-ear** |
| --- | --- | --- |
| Location (latitude, longitude) | -37.910799, 145.134639 | -37.909887, 145.132784 |
| Recorded Month/Year | January 2019 | November 2019 |
| Time recorded | $10 am-1 pm$ | $11 am-2 pm$ |
| Ambient temperature | $23 ^{\circ}C-26 ^{\circ}C$ | $22 ^{\circ}C-26 ^{\circ}C$ |
| Wind speed | $9 - 26 {kmh}^{-1}$ | $15 - 28 {kmh}^{-1}$ |
| Average distance from the ground cover | $60 cm$ | $95 cm$ |
| Average area covered by honeybee | $1465 \pm531$ pixels | $321 \pm273$ pixels |
| Number of flowers/inflorescences | 446 flowers | 48 inflorescences |
| Floral/Inflorescence density | $\sim2340 flowers m^{-2}$ | $\sim180 inflorescences m^{-2}$ |
| Size of the training dataset | 2799 images | 451 images |
| Number of ‘bees’ in images | 3706 | 728 |

**References**

[1] Tzutalin. LabelImg. 2019. Available: https://github.com/tzutalin/labelImg
